# Supplementary material for: Improved protocol for single-nucleus RNA-sequencing of frozen human bladder tumor biopsies
Source: Nucleus. 2023 Mar 6;14(1):2186686. doi: 10.1080/19491034.2023.2186686 (PMC10012951; doi:10.1080/19491034.2023.2186686)
Supplement: Supplemental Material [file KNCL_A_2186686_SM1479.zip › Supplementary table 2 (1).docx]

Supplementary table 2

| **Name/Oligo** | **Sequence** | **Conc.** | **Source** |
| --- | --- | --- | --- |
| Macosko-2011-10(V+) beads | 5’- Toyopearl-linker-beads-TTTTTTTAAG CAG TGG TAT CAA CGC AGA GTAC JJJJJJJJJJJJ NNNNNNNN V TTTTTTTTTTTTTTTTTTTTTTTTTTTTTT-3’ | 300,000 beads/μl | ChemGenes |
| TSO | AAG CAG TGG TAT CAA CGC AGA GTG AAT rGrGrG | 50 μM | IDT |
| SMART PCR Primer | AAG CAG TGG TAT CAA CGC AGA GT | 100 μM | IDT |
| New-P5-SMART PCR hybrid oligo | AAT GAT ACG GCG ACC ACC GAG ATC TAC ACG CCT GTC CGC GGA AGC AGT GGT ATC AAC GCA GAG T*A*C | 10 μM | IDT |
| Read 1 Custom SeqB | GCC TGT CCG CGG AAG CAG TGG TAT CAA CGC AGA GTA C | 50 μM | Sigma Aldrich |
| Nextera_N701 | CAA GCA GAA GAC GGC ATA CGA GAT **TCG CCT TA**G TCT CGT GGG CTC GG | 10 μM | Sigma Aldrich |
| Nextera_N702 | CAA GCA GAA GAC GGC ATA CGA GAT **CTA GTA CG**G TCT CGT GGG CTC GG | 10 μM | Sigma Aldrich |
| Nextera_N703 | CAA GCA GAA GAC GGC ATA CGA GAT **TTC TGC CT**G TCT CGT GGG CTC GG | 10 μM | Sigma Aldrich |
